# The convergence of genomic medicine and translational omics in transforming breast cancer patient care

**DOI:** 10.1172/JCI187520

**Published:** 2024-11-01

**Authors:** Sulin Wu, Yonglan Zheng, Olufunmilayo I. Olopade

**Affiliations:** 1 Section of Hematology and Oncology, Department of Medicine and; 2 Center for Clinical Cancer Genetics & Global Health, Department of Medicine, The University of Chicago, Chicago, Illinois, USA.

## Introduction

Breast cancer is the most diagnosed cancer among women worldwide, with an estimated 2.3 million new cases and 670,000 deaths reported in 2022 (1). Advances in genomic research have heralded a new era in precision medicine, enabling personalized treatment and risk assessment based on molecular profiles. Next-generation sequencing and deep-learning (DL) algorithms have transformed breast cancer care by facilitating the analysis of complex and extensive datasets, with the potential to democratize access to omics-informed clinical trials.

A comprehensive understanding of the heterogeneity and complexity of breast cancer across diverse populations, along with intricate disease mechanisms, is fueling innovations aimed at personalizing breast cancer care and reducing global disparities in outcomes.

## Navigating heterogeneity to optimize therapeutics

For decades, the treatment of breast cancer has been based on classification according to estrogen receptor (ER), progesterone receptor (PR), and human epidermal growth factor receptor 2 (HER2) status (2–5). However, advances in genomics technologies have underscored the critical role of molecular profiling in determining prognosis and guiding treatment strategies. For patients with early stage breast cancer, genomic information enables more accurate predictions of recurrence and metastasis risk, identifies molecular subtypes of the disease, and helps prevent overtreatment by refining traditional therapeutic approaches (Table 1). Furthermore, discovering pathogenic variants in BRCA1 DNA repair associated (*BRCA1*) and *BRCA2* genes has been pivotal to our understanding of both hereditary and sporadic breast cancers. These variants largely increase breast and ovarian cancer risks and identify patients who may benefit from targeted therapies like poly (ADP-ribose) polymerase (PARP) inhibitors. The OlympiA trials have shown that olaparib effectively reduces recurrence risk in patients with *BRCA*-mutated breast cancer, an example of enhanced outcomes through personalized care for high-risk individuals (6). For hormone receptor–positive (HR^^+^^) breast cancers, the addition of CDK4/6 inhibitors to hormonal therapies offers a more potent treatment, particularly for patients with high disease burden or progression on prior endocrine therapy. Selective ER degraders are a class of drugs representing a substantial advance in treating HR^^+^^ breast cancers by effectively degrading ER and inhibiting tumor growth. In contrast, HER2-positive breast cancers, known for their aggressive behavior, respond favorably to targeted therapies such as trastuzumab, pertuzumab, and tyrosine kinase inhibitors.

Recent innovations in antibody-drug conjugates (ADCs), such as trastuzumab emtansine (T-DM1) and trastuzumab deruxtecan (T-DXd), have notably improved survival outcomes for HER2-positive patients (7, 8). The FDA approval of T-DXd for unresectable or metastatic HER2-positive cases underscores the critical role of genomic and molecular profiling in treatment decisions. While these advances herald a promising future for breast cancer therapy, especially for a recently defined subset of HER2-low breast cancer, the challenge of broadening access to these drugs for rare subtypes of breast cancer remains. Triple-negative breast cancer (TNBC) is a particularly aggressive disease with limited treatment options. Immunotherapeutic agents like pembrolizumab have expanded treatment possibilities for PD-(L)1-positive TNBC, offering new hope for patients, though further research is needed to fully evaluate their impact (9). The development of Trop2-based ADCs also holds promise for improving TNBC outcomes (10). Applying artificial intelligence (AI) and DL algorithms to analyze the tumor microenvironment (TME) can substantially enhance ADC development by identifying therapeutic targets and optimizing antibody design. Integrating genetic, pharmacogenomic, and histopathologic data can further improve the selection of antibody targets and cytotoxic payloads, making ADCs more versatile therapeutic options across multiple cancer types.

## Precision medicine for breast cancer

Precision oncology integrated with germline genetic testing has transformed breast cancer care by revealing the genetic underpinnings of disease development, recurrence risk, and treatment resistance. These advances facilitate more personalized cancer treatments tailored to each patient’s unique genetic and molecular profiles.

A milestone in the molecular characterization of breast cancer was the discovery of the *BRCA1* and *BRCA2* genes. *BRCA1* pathogenic variants, enriched in basal-like/TNBC, and the epigenetic silencing of *BRCA1*, which impairs DNA repair, play significant roles in cancer progression (11). The advancement of polygenic risk scores (PRSs) based on breast cancer genetic variants identified by GWASs allows for more personalized breast cancer screening, which supports early detection and efficient prevention strategies (12–14). Integrating genomic, epigenomic, and pseudogene expression data with clinical features holds promise for developing precise prognostic models to personalize treatment and accelerate progress in prevention and early detection, with potential to reduce disparities in breast cancer outcomes when appropriately deployed for population health management (15).

Given the paucity of omics data from non-European ancestry populations, large datasets from initiatives such as The Cancer Genome Atlas and the International Cancer Genome Consortium have provided comprehensive catalogs of genomic aberrations that inform clinical management and precision medicine. The genomic landscape of breast cancer is notably heterogeneous, including mutations, copy number variations, and structural alterations that affect tumor progression and treatment response (16). Advances in high-throughput sequencing and computational biology have identified differences in genomic alterations associated with aggressive breast cancer progression and metastasis in diverse populations. Notable findings in the mutational landscape include the mutually exclusive presence of *GATA3* and *TP53/PIK3CA* pathogenic variants, higher levels of intratumoral heterogeneity, and genomic instability in breast cancer among Nigerian women, as well as the discovery of a unique breast cancer subtype defined by early clonal pathogenic variants in *GATA3* and a younger age at diagnosis (17). The *TP53* gene is the most frequently mutated in breast cancer, particularly in TNBC and HER2-positive subtypes. Patients with *TP53*-mutant breast cancer typically face a higher risk of recurrence, poorer overall survival, and a greater likelihood of metastasis compared with those with wild-type *TP53*. There is an urgent need to identify upstream lifestyle and environmental risk factors for specific mutational signatures. Various clinical trials currently explore WEE1 and MDM2/MDMX inhibitors for *TP53*-mutated cancers. Additionally, *PIK3CA* pathogenic variants, common in HR^^+^^ breast cancers, activate the PI3K/AKT/mTOR pathway and are effectively targeted by alpelisib combined with endocrine therapy. Mutations in other genes, such as *PTEN*, *AKT1*, and *ESR1*, guide the development of novel inhibitors and combination therapies to overcome resistance mechanisms.

While genomics offers insights into mutations and genetic variability, proteomics translates these changes into the biological mechanisms that drive cancer progression, drug resistance, and treatment response. Initiatives like the Clinical Proteomic Tumor Analysis Consortium significantly enhance our understanding of genetic alterations at the protein level. Integrative approaches, including digital pathology, provide a comprehensive view of the molecular landscape, fostering the creation of highly effective, individualized treatment strategies. Modern techniques now allow for the simultaneous assessment of multiple biomarkers, revealing complex interactions within the TME. These advances will be crucial for developing personalized treatments, deepening our understanding of cellular diversity and refining prognostic and predictive assessments, ultimately improving therapies and biomarker development in the future (18).

## Advancing biomarker development with AI and DL

AI-based imaging algorithms and DL models enhance the accuracy and efficiency of cancer detection, assisting radiologists in identifying breast lesions (19). AI also automates breast-density assessments, enabling earlier diagnosis and intervention. By integrating imaging data with patient-specific omics profiles, computational methods can aid in developing personalized treatment plans (20). AI could offer high accuracy in classifying cancerous tissues, underscoring its critical role in diagnostic precision and tailored treatments (21). Furthermore, radiogenomics links imaging features with genetic mutations to predict molecular pathway activity and optimize treatment. For example, tumor texture heterogeneity on imaging can indicate *ERBB2* amplification, guiding the use of trastuzumab or pertuzumab and highlighting the importance of the TME.

The TME, composed of a diverse array of cell types in a dynamic and intricate structure surrounding the tumor, plays a critical role in immune modulation, making it a crucial target for advancing more effective immunotherapeutic strategies. Alterations in the TME can influence the release of tumor-derived materials, including circulating tumor cells, cell-free DNA, and exosomes, into the bloodstream. Analyzing these components provides valuable insights into tumor dynamics and treatment response. Liquid biopsy, utilizing advanced cell identification and single-cell sequencing, serves as a noninvasive tool to track disease progression and detect resistance mutations with high sensitivity and specificity. For example, detecting *ESR1* mutations in HR^^+^^ breast cancer can predict resistance to aromatase inhibitors, guiding a switch to therapies such as fulvestrant.

## Challenges and opportunities

Breast cancer presents a complex landscape for precision medicine, with challenges arising from the disease’s intricacies and the advanced technologies required to address them. Precision medicine, grounded in genetic insights, offers promise, through integrating germline and somatic profiling to guide treatment decisions. Additionally, genetic testing raises ethical concerns, such as incidental findings and potential privacy issues, requiring education for healthcare providers and an informed public to participate in innovative clinical trials.

Despite these challenges, AI-based integrative research and advanced computational methods offer potential approaches to identify novel biomarkers and optimize breast cancer treatment regimens. Innovative clinical trial designs, such as platform and basket studies, enable validation of these biomarkers. Technologies such as single-cell RNA sequencing provide deeper insights into TME heterogeneity and resistance mechanisms. The convergence of AI, genomics, immunotherapy, and digital pathology is set to revolutionize breast cancer treatment, paving the way for more personalized and effective therapies. Key to our success will be high quality cancer care powered by affordable access to health insurance, innovative clinical trial design, and community-engaged research so that every person has a fighting chance to survive and thrive after a diagnosis of breast cancer, the most common female cancer in the world.

## Figures and Tables

**Table 1:**
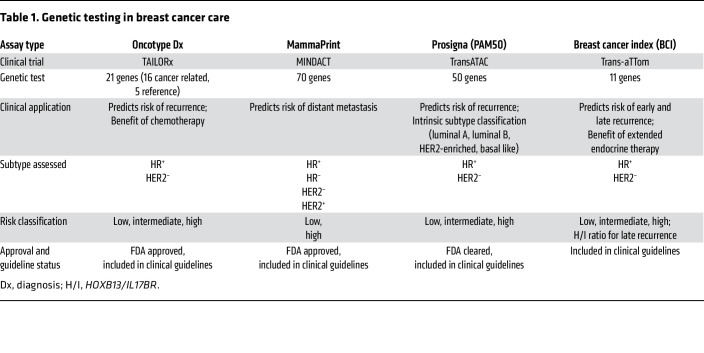
Genetic testing in breast cancer care

## Version 1 11/01/2024

Electronic publication
